# Supplementary material for: Population Genomics Study and Implications for the Conservation of Zabelia tyaihyonii Based on Genotyping-By-Sequencing
Source: Plants (Basel). 2022 Dec 30;12(1):171. doi: 10.3390/plants12010171 (PMC9823854; doi:10.3390/plants12010171)
Supplement: Supplementary file 1 [file plants-12-00171-s001.zip › supplementary files.pdf]

Supplementary material

	SC	BS	CW	TG	YC	SH
SC		1.606	1.156	1.092	1.314	0.522
BS	0.135		2.220	2.162	3.217	0.779
CW	0.178	0.101		2.346	1.838	0.640
TG	0.186	0.104	0.096		1.834	0.591
YC	0.160	0.072	0.120	0.120		0.725
SH	0.324	0.243	0.281	0.297	0.256	

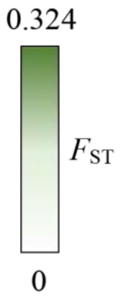

Figure S1. Genetic differentiation coefficient F_{ST} (below diagonal) and gene flow *Nm* (above diagonal) between populations. All F_{ST} values significantly differed from zero after Bonferrooni adjustments (P < 0.003)

Table S1. The sampling information of six populations of *Zabelia tyaihyonii*

Population	Number of Individuals	Collection Locality	Coordinates	Altitude
**SC**	14	Sincheon-ri, Yeongwol-gun, Gangwon-do province, South Korea	N37.24072 E128.31089	270m
**BS**	21	Bukssang-ri, Yeongwol-gun, Gangwon-do province, South Korea	N37.20327 E128.40826	270m
**CW**	18	Changwon-ri, Yeongwol-gun, Gangwon-do province, South Korea	N37.17003 E128.33855	337m
**TG**	14	Togyo-ri, Yeongwol-gun, Gangwon-do province, South Korea	N37.16549 E128.34287	289m
**YC**	14	Yeongcheon-ri, Danyang-gun, Chungcheongbuk-do province, South Korea	N37.06122 E128.30331	200m
**SH**	13	Sinhyeon-ri, Jecheon-si, Chungcheongbuk-do province, South Korea	N36.92325 E128.15417	254m
